# Bromelain Supplementation in the Management of Otitis Media with Effusion in Children

**DOI:** 10.3390/children11121440

**Published:** 2024-11-26

**Authors:** Francesco Martines, Ginevra Malta, Emanuele Cannizzaro, Theodoridou Kelly, Pietro Salvago, Fulvio Plescia

**Affiliations:** 1 Department of Biomedicine, Neuroscience and Advanced Diagnostics (BiND), Section of Audiology, University of Palermo, Via del Vespro 129, 90127 Palermo, Italy; francesco.martines@unipa.it (F.M.); pietro.salvago01@unipa.it (P.S.); 2 Department of Health Promotion Sciences, Maternal and Child Care, Internal Medicine and Medical Specialties ‘Giuseppe D’Alessandro’, University of Palermo, Via del Vespro 133, 90127 Palermo, Italy; ginevra.malta@unipa.it (G.M.); emanuele.cannizzaro@unipa.it (E.C.); 3 Department of Microbiology, Andreas Syggros University Hospital Athens Greece, 10552 Athens, Greece; ktheodoridou@med.uoa.gr

**Keywords:** bromelain supplement, otitis media with effusion, tympanometry, otoacoustic emissions, audiometry, otoscopy

## Abstract

Background/Objectives: The respiratory system is prone to infectious diseases, especially in children below five years of age. Upper respiratory tract infections in children are often associated with Eustachian tube dysfunction and complicated by otitis media with effusion (OME), an inflammatory process within the middle ear, which can lead to hearing loss. Treatment for these infections involves a combination of medication and symptom relief, depending on the severity and cause of the infection. In recent years, natural therapeutic drugs derived from herbal medicines have been gaining popularity in treating various pathologies. Bromelain, one of the most studied natural compounds, has been investigated extensively due to its numerous pharmacological properties, offering a potential new avenue for treatment. Based on these promising findings, our study was designed to examine the efficacy of supplementation with bromelain in countering symptoms associated with OME. Methods: This study was conducted on data acquired from medical records from the Section of Audiology of the University of Palermo, focusing on the period of January 2022 to June 2023 and selecting 224 children (age range 1–8 years), namely 174 males and 50 females, who were evaluated for presumed OME at the audiology pediatric ambulatory. All patients selected before initiating pharmacological treatment underwent thorough screening regarding the functionality of the tympanic cavities, otoacoustic emissions, the auditory threshold, and the ear canal’s integrity. Results: The preliminary findings of this study are significant, demonstrating that supplementation with bromelain led to notable improvements in the symptoms accompanying OME after 15 days and 60 days of therapy. Notably, patients who received the bromelain supplement reported reduced mucus secretions and improved auditory function. Conclusions: These results underscore the potential of naturally occurring compounds as adjuvants to standard therapeutic strategies in treating OME.

## 1. Introduction

The respiratory system is highly susceptible to infectious diseases due to the broad and easy access for foreign agents. It is divided into the upper and lower airways, each containing numerous subsections [1]. The upper respiratory tract (URT) consists of the nasal cavity, pharynx, and larynx, which are commonly affected by infections such as nasopharyngitis, pharyngitis, and tonsillitis [2,3]. Interestingly, upper airway infections represent one of the top three diagnoses in the outpatient setting and mainly affect children under five years of age and those attending children’s communities [4,5]. In this part of the population, inflammation caused by URT infections can cause drainage problems within the sinuses or middle ear, creating significant potential for secondary bacterial infections such as sinusitis or otitis media (OM) [6,7].

OM is classified into two types: acute OM and OM with effusion (OME). Acute OM involves the sudden onset of symptoms such as ear pain, fever, and hearing loss that last for less than six weeks. This is the most common form in young children and is usually caused by a bacterial or viral middle ear infection that typically accompanies an upper airway infection, distinguished by ear pain, hearing disorder, fever, and otorrhea [8]. OME is a condition involving fluid collection in the middle ear, associated with either a mild or moderate conductive hearing impairment. It is caused by several factors, including structural issues with the eustachian tube, recurrent acute OM, or persistent exposure to environmental irritants such as tobacco smoke or air pollution [9,10,11].

In children, treating the OM disease involves a combination of medication and symptom relief and depends on the infection’s cause and severity. Usually, decongestants, pain relievers, mucolytic drugs, and antibiotics are used to alleviate symptoms and treat the underlying condition [12,13]. Although these drugs provide relief to the patient, they are certainly not free of side effects and sometimes can lead to therapeutic failure. For example, the extensive use of antibiotics can lead to resistance, which, in children, is a growing concern in the medical community [14]. It can become difficult or impossible to treat some infections due to this, leading to longer recovery times. Moreover, OME in children can result in specific complications, impairing the child’s language and behavioral development. Therefore, the identification of new therapeutic candidates and innovative alternative therapies to treat OME is essential, as timely and proper treatment can help to prevent these complications and improve the outcomes.

Natural therapeutic drugs derived from herbal medicines have garnered significant attention in the medical community recently. Bromelain is one of the most extensively studied natural compounds thanks to its diverse pharmacological properties [15,16,17]. Bromelain, an enzyme naturally found in fresh pineapple fruit, contains a complex mixture of different thiols and other endopeptidases, including various protease inhibitors, glucosidase, cellulase, phosphatase, peroxidase, and escharase [18,19]. As a phytopharmaceutical compound, bromelain has demonstrated several beneficial properties [20]. These include fibrinolytic, proteolytic, antithrombotic, antifungal, and antibacterial actions [21,22,23]. Importantly, there is evidence for its potential anti-cancer activity [24], which could have significant implications for research or clinical practice.

Bromelain has been found to play a pivotal role in modulating the inflammatory response [25,26]. Specifically, it acts by inhibiting the synthesis of pro-inflammatory prostaglandins while leaving the expression of the anti-inflammatory ones unaffected [17,18,27,28]. This unique mechanism allows bromelain to restore the balance between the prostaglandins, a critical factor in maintaining a healthy organism. These anti-inflammatory properties could reduce swelling in acute sinusitis, sore throat, arthritis, and gout and accelerate recovery from injuries and surgery [20]. This promising potential could open new avenues for the treatment of various health conditions, sparking optimism in the medical community.

Based on the above premise, the present retrospective study sought to understand whether supplementation with a natural dietary supplement containing bromelain 400 mg could act synergistically with the commonly adopted standard therapy to reduce OME signs such as eardrum movement, the functionality of the cochlear outer hair cells and hearing thresholds, and mucus secretion. This study provides additional information regarding the therapeutic efficacy of supplementation with naturally derived supplements and offers new insights for basic and clinical research.

## 2. Materials and Methods

### 2.1. Study Sample

The present study evaluated the therapeutic efficacy of administering a natural bromelain 400 mg (1000 GDU/g) supplement in treating OME. This study was conducted on data acquired from medical records from the Section of Audiology of the University of Palermo, selecting patients treated from January 2022 to June 2023, including 224 children (age range 1–8 years), namely 174 males and 50 females (M/F ratio = 3.48), who were evaluated for suspected OME at the audiology pediatric ambulatory. All procedures were conducted following the ethical principles of the Declaration of Helsinki. The parents of the participating children signed a written informed consent form, authorizing the possible anonymous data acquisition for scientific studies.

This study included children with OME who had not undergone ear surgery previously. Children with a prior diagnosis of otitis media or who had received treatment for a URT infection within six months were excluded to prevent any potential influence of the prior treatment on the study results. The criteria for the diagnosis of OME were as follows: a documented middle ear effusion and/or air fluid bubble by otoscopic examination in the presence of B or C tympanograms and conductive hearing loss greater than 25 dB at any one of the frequencies from 250 Hz through 4 kHz [5].

The selected sample was divided into the natural supplement group (NSG) and the control group (CTG). The NSG comprised those who received 400 mg of bromelain (1000 GDU/g), administered in syrup form, in combination with the standard treatment, while the CTG received the standard treatment only. The standard treatment consisted of nasal irrigation with fluticasone, a synthetic glucocorticoid commonly used as an anti-inflammatory medication due to its capability to reduce inflammation in the nasal passages, and a hypertonic saline solution, typically used to manage otitis media.

**Figure 1:**
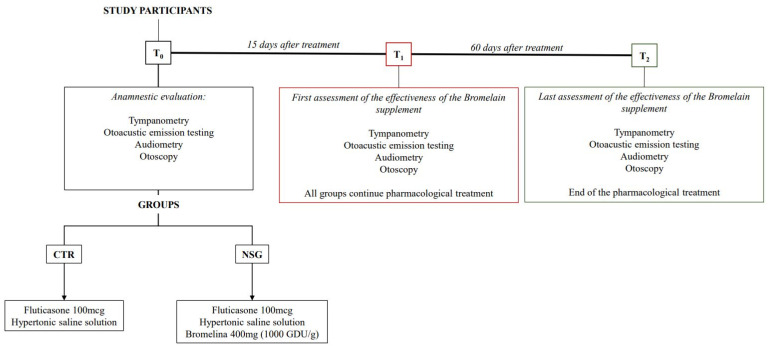
Timeline. CTR: control group; NSG: natural supplement group. T0 (anamnestic evaluation): initial assessment of OME diagnosis and audiometry function before any treatment; T1 (15 days after the beginning of the pharmacological treatment): first follow-up to evaluate the efficacy of treatment, including tympanometry, otoacoustic emission, audiometry, and otoscopy; T2 (end of the pharmacological therapy at 60 days): final follow-up assessment to compare the results with T0 and T1, measuring the progression or resolution of OME symptoms and auditory function.

The children belonging to the NSG took 5 mL of syrup containing 400 mg of bromelain (1000 GDU/g) daily for two months, with the supplement taken between meals to ensure optimal absorption, as food may interfere with its efficacy. The diagnostic evaluations performed to diagnose and assess the possible percentage of healing following drug therapy were the presence of mucus in the ear canal, tympanograms, otoacoustic emissions, and audiometric evaluations. These parameters were evaluated via diagnostic evaluations performed before the start of treatment (T0), 15 days after the treatment (T1), and at the end of the observational period (T2) (Figure 1).

### 2.2. Tympanometry

Tympanometry is a diagnostic test that evaluates middle ear function by measuring the eardrum’s movement in response to changes in air pressure. A small probe is inserted into the ear canal to assess the eardrum’s reaction, resulting in a tympanogram that indicates the eardrum and middle ear’s compliance. This test helps to identify issues like fluid or obstructions that could affect hearing. It is typically quick and painless, although it may be cause discomfort if the eardrum is inflamed or infected.

Tympanometry was performed with a Flute Diagnostic Middle Ear Analyzer system (Inventis, Padua, Italy) using the standard settings and a 226/1000 Hz probe tone (depending on the age), with an air pressure range of −400 to +200 daPa, with automatic recording. Three different functional curves for middle ear structure motility were obtained from the test: tympanogram type A outlined typical function; tympanogram type B pointed out the presence of a phlogistic process in the middle ear, which interferes with auditory transmission; tympanogram type C showed negative pressure in the middle ear, as in the case of eustachian tube dysfunction or nasopharyngeal obstruction. The results of the acoustic impedance test were associated with the following scores: 1 = type A tympanogram, 2 = type B tympanogram, and 3 = type C tympanogram. They were analyzed for each ear.

### 2.3. Otoacoustic Emissions

Transient-evoked otoacoustic emissions (TEOAEs) were recorded using the Otodynamics ILO 288 USB II device with the standard settings; the stimulus was a nonlinear click at an intensity of 84 dB SPL, and a total of 260 averages was used. TEOAEs were considered “PASS” only when the reproducibility of the recorded emissions exceeded 70% in at least three octave bands and the stimulus stability exceeded 80%. A second TEOAE test was also performed in re-examined infants to see if the middle ear was disease-free. The following scores were assigned to the test results: 1 in the case of TEOAEs present (pass) and 2 in the case of the absence of TEOAEs (refer).

### 2.4. Audiometry

Hearing ability was assessed through an audiometric examination with a Piano Plus VRA clinical audiometer (Inventis, Padua, Italy). This non-invasive examination allowed us to distinguish the various types of hearing loss. The test was conducted in a sound-proofed room; hearing threshold levels were measured in the sound field, under earphones or through a bone conduction transducer according to the patient’s age. Pure-tone air conduction (AC) and bone conduction (BC) thresholds were investigated for each ear at frequencies of 0.5, 1, 2, and 4.0 kHz. Sound field testing was performed if the child was not amenable to wearing headphones and binaural testing was performed in all other children. The pure-tone average (PTA) was calculated by the averaging of the AC threshold levels of 0.5, 1, 2, and 4 kHz.

Four different degrees of hearing loss were analyzed: mild hearing loss (hearing threshold between 20 and 40 decibels)—the patient perceived most speech, but gentle and light sounds were difficult to hear, especially in noisy situations; moderate hearing loss (40–70 dB)—the patient had great difficulty understanding a person who was speaking at a normal tone of voice; severe hearing loss (70–95 dB)—patients did not understand what a person was saying at an average voice level, and they could hear only a few loud sounds; profound hearing loss (>96 dB)—the patient did not perceive any speech, but only a few thunderous sounds. In this case, comprehension was achieved almost solely through lip reading and sign language or with an implant.

The following scores were assigned to the different degrees of hearing loss: 1 = mild hearing loss (20–40 dB); 2 = moderate hearing loss (40–70 dB); 3 = severe hearing loss (70–95 dB); and 4 = profound hearing loss (>96 dB).

### 2.5. Otoscopy

The middle ear was studied through a video-otoscopic examination. This assessment was carried out because the presence and type of secretions within the middle ear usually indicate prolonged inflammation with a feeling of ear clapping that, in the child, generates transient hearing loss and confusion.

This condition is associated with an alteration in mucociliary transport function, and the Eustachian tube’s function involves the accumulation of fluid in the tympanic cavity that can be identified as serous, mucous, or seromucous. The absence of mucus (typical picture) was identified by a score with a value of one (score = 1); the various types of mucus were assigned the following scores: serous = 2; mucous = 3; seromucous = 4.

### 2.6. Statistical Analysis

Data were analyzed using the GraphPadPrism 8.01 statistical software package (GraphPad Company, San Diego, CA, USA). Initially, the collected data were analyzed to understand whether they were distributed normally or not, so as to choose the most appropriate statistical analysis to apply. To do this, we applied the Kolmogorov–Smirnov normal distribution test. Considering that our data followed a normal distribution for some tests, while others had a non-normal distribution, we used the tests below.

Following a normal distribution, the data obtained from the tympanometric examination and the TEOAE test in the follow-up time were analyzed using a two-way ANOVA for repeated measurements, followed by a post hoc Bonferroni test with an ordinary alpha = 0.5. The data were SEM averages of the scores obtained from the various tests. Statistical analysis was considered significant for *p* < 0.05. As for the data obtained from scores obtained with audiometry, since these followed a non-normal distribution, they were analyzed through the Kruskal–Wallis test followed by Dunn’s multiple comparison post-test. A chi-squared test was also conducted to determine whether there were any differences in the scores obtained from the various examinations conducted between the patients in the two test groups in the two follow-up times.

The Pearson r test was employed to analyze the correlations between fluid accumulation in the tympanic cavity and the parameters recorded through various instrumental investigations during the initial visit.

## 3. Results

### 3.1. Correlations

**Figure 2:**
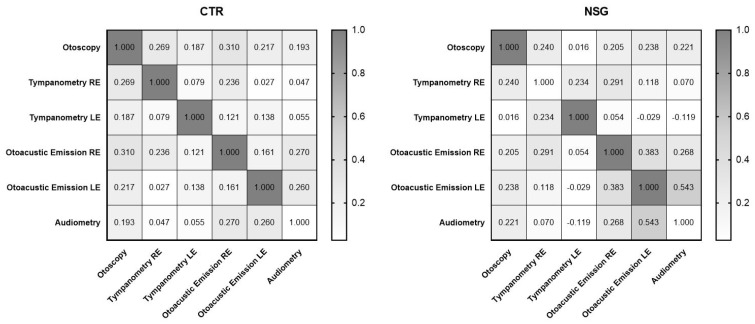
The correlation matrix shows how fluid in the tympanic cavity correlates with middle ear function detected by tympanometry, otoacoustic emissions, and the auditory threshold detected by audiometry. CTG, control group; NSG, natural supplement group; RE, right ear; LE, left ear.

The data obtained from the video-otoscopic evaluation were compared to the data obtained from the other instrumental analyses before the start of the pharmacological treatment (T0). The statistical analysis, conducted through the Pearson r test, showed a positive correlation between the fluid in the tympanic cavity and the auditory deficit recorded via tympanometry evaluation and otoacoustic emissions in patients assigned to both experimental groups. Furthermore, the results indicated a positive correlation between secretions in the tympanic cavity and auditory deficits detected by the audiometric examination in both groups (Table 1 and Figure 2). These findings highlight the importance of regular check-ups to detect and address any potential issues with hearing health.

### 3.2. Tympanometry

**Figure 3:**
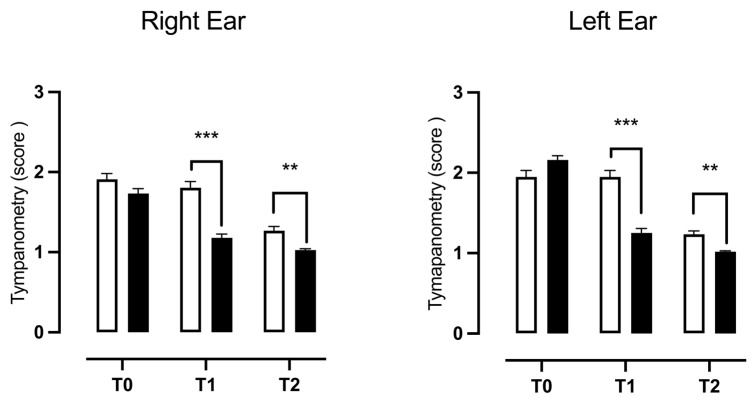
Differences in middle ear function, evaluated during follow-up periods, between patients treated with standard therapy plus 400 mg bromelain and those who received only standard therapy. The data refer to 224 patients divided into a control group (□ CTG) *n* = 112 and natural supplement group (■ NSG) *n* = 112; the data are represented as the SEM averages of the scores obtained from the tympanogram. *** *p* < 0.001; ** *p* < 0.01 vs. CTG. T0: baseline; T1: first check visit; T2: second check visit.

To assess possible improvements in middle ear function and the mobility of the tympanum and ossicle chain due to the prescribed therapy, an impedance test was conducted on all patients during the two follow-ups (T1 and T2). The results were compared to each other and with those obtained during the first visit (T0). The results obtained from the two-way ANOVA for repeated measurements showed a significant effect of the treatment, time, and their interaction on the score obtained from the impedance test, both in the right (F(1, 222) = 37.72, *p* < 0.0001; F(2, 444) = 106.9, *p* < 0.0001; F(2, 444) = 13.91, *p* < 0.0001) and in the left (F(1, 222) = 11.48, *p* = 0.0008; F(2, 444) = 199.77, *p* < 0.0001; F(2, 444) = 44.62, *p* < 0.0001) ear. In detail, the post hoc analysis conducted with the Bonferroni multiple comparison post-test highlighted a significant improvement in the right and left tympanogram both at T1 (t = 6.765, *p* < 0.0001; t = 7.128, *p* < 0.0001) and T2 (t = 3.457, *p* = 0.0023; t = 3.142, *p* = 0.0062) in the NSG compared to the CTG (Figure 3). A chi-squared test was also conducted to assess the percentage of subjects who had recovered after the addition of the dietary supplement to the prescribed drug therapy. The data analysis showed a significant increase in subjects with type A tympanograms and a sharp reduction in the number of subjects diagnosed with type B tympanograms in the NSG compared to the CTG. There was also a significant reduction in the NSG patients with C-type tympanogram diagnoses for the left ear (Table 2).

### 3.3. Otoacoustic Emissions

All patients were examined for TEOAEs to assess the correct functionality of the external cochlear ciliary cells. The examination was repeated in all patients in the two follow-up periods (T1 and T2), and the results obtained were compared with those obtained during the first examination (T0).

**Figure 4:**
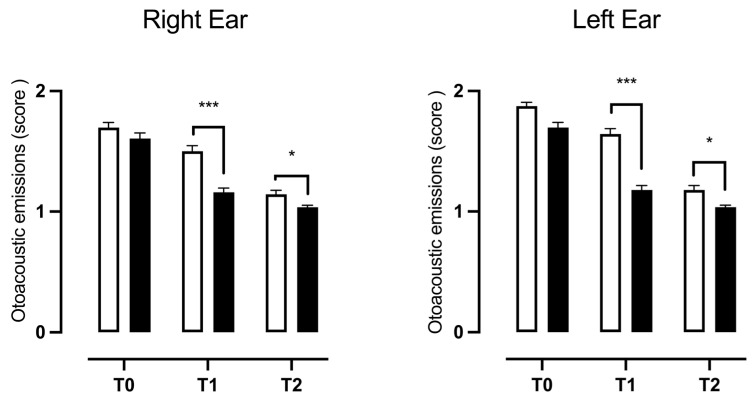
Differences in TEOAEs between patients treated with standard therapy plus 400 mg bromelain and those treated with standard therapy. The data refer to 224 patients divided into a control group (□ CTG) *n* = 112 and a natural supplement group (■ NSG) *n* = 112; the data are represented as the SEM averages of the scores obtained from the analysis of TEOAEs. *** *p* < 0.001; * *p* < 0.05 vs. CTG. T0: baseline; T1: first check visit; T2: second check visit.

The results of the two-way ANOVA for repeated measurements considering the treatment with “the supplement” as a variable between patients and the “timing” as a variable within patients showed a significant effect of the treatment, time, and their interaction on the score obtained in this examination, both for the right ear (F(1, 222) = 19.52, *p* < 0.0001; F(2, 444) = 158.27, *p* < 0.0001; F(2, 444) = 9.645, *p* < 0.0001) and for the left ear (F(1, 222) = 53.01, *p* < 0.0001; F(2, 444) = 229.4, *p* < 0.0001; F(2, 444) = 15.40, *p* < 0.0001). The data obtained through the Bonferroni multiple comparison post-test analysis showed that the addition of the natural supplement to the standard therapy was able to induce a significant improvement in the TEOAEs in both ears at T1 (t = 5.762, *p* < 0.0001; t = 7.974, *p* < 0.0001) and a further increase in patients with pass-type TEOEs for the right and left ears at time T2 (t = 2.850, *p* < 0.0148; t = 3.537, *p* < 0.0016) with respect to patients that received only the standard therapy (Figure 4).

A chi-squared test was also performed to assess the percentage of patients who moved from a condition without acoustic emissions to one with acoustic emissions due to dietary supplement administration. The data analysis showed a reduction in the number of patients without acoustic emissions in the NSG compared to the CTG (Table 3).

### 3.4. Audiometry

**Figure 5:**
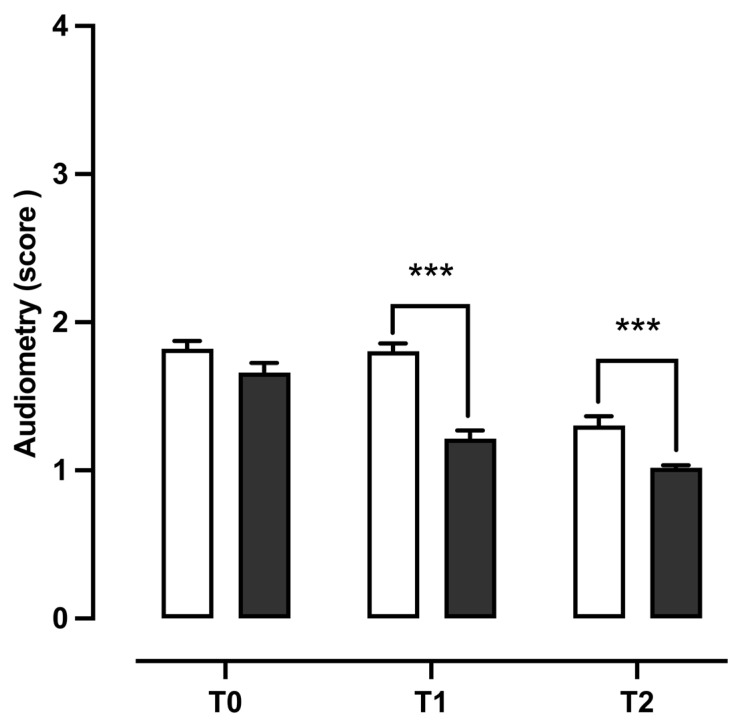
Differences in tonal audiometry between patients treated with standard therapy plus 400 mg bromelain and those treated with standard therapy. The data refer to 224 patients divided into a control group (□ CTG) *n* = 112 and a natural supplement group (■ NSG) *n* = 112; the data are represented as the SEM averages of the scores obtained from the analysis of tonal audiometry: 1 = mild hearing loss (20–40 dB); 2 = moderate hearing loss (40–70 dB); 3 = severe hearing loss (70–95 dB); and 4 = profound hearing loss (>96 dB). *** *p* < 0.001 vs. CTG. T0: baseline; T1: first check visit; T2: second check visit.

All patients were subjected to tonal audiometry to identify hearing problems. Thanks to this test, it was possible to determine the auditory threshold for each patient during the first visit (T0) and in the two follow-up times (T1 and T2). In particular, we evaluated the variation in hearing loss, which showed an improvement, while remaining, on average, within the range of mild hearing loss. The data analysis was conducted using a Kruskal–Wallis test, which revealed that there was a progressive improvement in hearing during the treatment period and between the groups (H(6, 672) = 255.3, *p* < 0.0001). In detail, Dunn’s multiple comparison post-test analysis showed a greater improvement in hearing ability in NSG patients than in CTG patients at both the T1 (*p* < 0.0001) and T2 (*p* < 0.0003) follow-up times (Figure 5).

### 3.5. Clinical Findings Observed During Otoscopy

**Figure 6:**
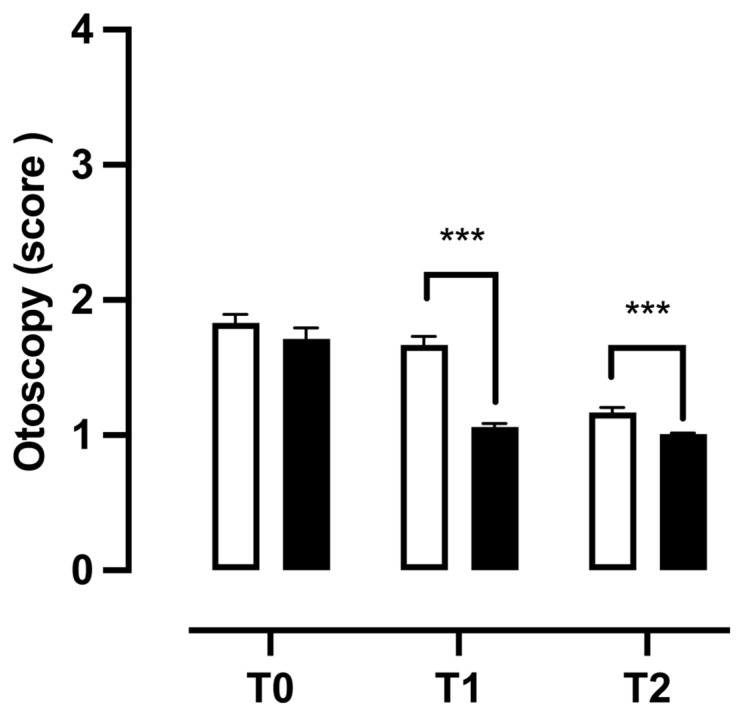
Differences in fluid secretion in the tympanic cavity between patients treated with standard therapy plus 400 mg bromelain and those treated with standard therapy. The data refer to 224 patients divided into a control group (□ CTG) *n* = 112 and a natural supplement group (■ NSG) *n* = 112; the data are represented as the SEM averages of the scores obtained from the analysis of tonal otoscopy. *** *p* < 0.001 vs. CTG. T0: baseline; T1: first check visit; T2: second check visit.

All patients underwent an otoscopic examination to assess whether 400 mg bromelain supplementation alongside the standard therapy used to resolve OME could modify the type of fluid secretions in the tympanic cavity. The results of the two-way ANOVA for repeated measurements considering the treatment with “the supplement” as a variable between patients and the “timing” as a variable within patients showed a significant effect of the treatment, time, and their interaction on the score obtained in this examination (F(1, 222) = 39.26, *p* < 0.0001; F(2, 444) = 101.91, *p* < 0.0001; F(2, 444) = 15.99, *p* < 0.0001). The data obtained through the Bonferroni multiple comparison post-test analysis showed that the addition of the natural supplement to the standard therapy was able to induce a significant reduction in mucous secretions at T1 and T2 (t = 9.499, *p* < 0.0001; t = 4.762, *p* < 0.0001) with respect to patients that received only the standard therapy (Figure 6).

## 4. Discussion

This study found that adding bromelain to the standard therapy commonly used to treat OME in children reduced the healing times in most of the treated population. The instrumental analysis at the first follow-up showed that the group receiving bromelain supplements in addition to standard therapy had a significantly lower rate of type B and type C tympanograms than those receiving the standard therapy alone with fluticasone and saline solution. Moreover, the number of children with pass-type otoacoustic emissions increased during both follow-up times when the natural compound was added to the standard therapy. This improvement in the otoacoustic emissions was directly linked to the enhancement in hearing observed during the audiometric evaluation of children in treatment with the addition of a bromelain supplement, compared to the control group.

Numerous scientific studies have shown that, during the acute phase of URT infections caused by viruses such as rhinoviruses, adenoviruses, respiratory syncytial viruses, and influenza viruses [29,30,31], the body’s immune system releases various inflammation mediators. Some of these include pro-inflammatory cytokines like interleukin-1 (IL-1), interleukin-6 (IL-6), and tumor necrosis factor-alpha (TNF-alpha), as well as prostaglandins, bradykinin, leukotrienes, and free radicals [32,33]. This inflammatory response is crucial in regulating the immune response in the middle ear, and it also triggers the cells lining the mucous membranes of the middle ear to secrete mucus, which acts as a crucial defense mechanism by trapping and fighting off the pathogens that cause URT infections.

When an individual experiences a prolonged or recurrent infection, such as OM, the balance between inflammation and mucus secretion in the body may become disrupted; this can cause structural changes in the body, including the enlargement of the mucous glands [34,35]. Such hypertrophy can contribute to the formation and buildup of mucus, which can then impact the anatomy and function of the eustachian tube, whose primary role is to ventilate the eardrum case and maintain the balance and pressure of the middle ear and the external environment. However, when mucus accumulates, the natural ventilation and drainage of the middle ear can become obstructed [36]. This blockage can impede the passage of sound to the inner ear, leading to temporary hearing loss. This can also affect children’s learning abilities and language articulation [37,38].

The improvement in the hearing abilities found in the group that took the natural supplement with the standard therapy can be attributed to the synergistic therapeutic effect played by fluticasone, whose role in inflammatory processes is widely demonstrated [39], and by bromelain.

Bromelain is a natural substance that has been found to possess a range of remarkable health benefits for humans. This powerful enzyme has been shown to positively impact the production and release of cytokines, essential in fighting inflammation and supporting the immune system. Different studies have shown that bromelain can exert anti-inflammatory effects through multifactorial mechanisms involving the modulation of different molecular signaling pathways underlying the genesis of inflammation in different diseases, such as arthritis, asthma, and cancer [18]. One of the main anti-inflammatory mechanisms of bromelain is the inhibition of the signal transduction pathway mediated by nuclear factor-kappa B (NF-κB). This factor, under normal conditions, is present at the cytoplasmic level, linked to a complex of inhibitory proteins of the IκB family, which prevent NF-κB from migrating towards the nucleus and binding to specific sequences (κB) within target gene promoters and regulate the transcription of pro-inflammatory genes, including cytokines, chemokines, and adhesion molecules. During the inflammatory process, the production of cytokines such as TNF-alpha and IL-1 promotes IκB kinase (IKK) activation, which phosphorylates IκB, allowing NF-κB to translocate into the nucleus and proceed with the transcription activation of pro-inflammatory genes [40,41]. Bromelain acts precisely at this site by reducing the phosphorylation of IκB and the consequent stabilization of the protein. All of this leads to a reduction in the effect of NF-κB, with a resulting decrease in the production of key pro-inflammatory cytokines such as TNF-alpha, IL-1, and IL-6, as well as reducing the expression of cyclooxygenases (COX2) and cell adhesion molecules (ICAM-1, VCAM-1) [42]. In addition, bromelain promotes the expression of IL-10, an anti-inflammatory cytokine that plays a crucial role in reducing inflammation through the inhibition of the synthesis of pro-inflammatory cytokines and suppressing excessive immune responses, preventing excessive tissue damage and improving the body’s ability to heal and recover from injury or disease [43].

Bromelain can reduce the inflammatory response by directly inhibiting the expression of cyclooxygenases (COX), particularly COX-2. These enzymes are responsible for the synthesis of prostaglandins from arachidonic acid, leading to the production of pro-inflammatory prostaglandins E2 (PGE2) and thromboxane A2. Bromelain also increases the levels of cyclic platelet adenosine monophosphate, which, in turn, promotes a reduction in the activation of inflammatory cells and promotes the synthesis of prostaglandins PGI2 and PGE1 [44], which are crucial for tissue healing. Moreover, bromelain’s downregulation of PGE2 by inhibiting NF-κB and COX-2 could regulate the overexpressed immunogenic pathways and reduce lung inflammation [45,46]. Furthermore, bromelain limits cell migration immunity by limiting neutrophil migration to inflamed areas. To do this, it removes several cell surface molecules, including CD128a/CXCR1, CD128b/CCRC2, CD14, CD44, CD16, and CD 21, which are involved in leukocyte trafficking, cellular adhesion, and the induction of pro-inflammatory mediators. Researchers have shown that bromelain reduces P-selectin-mediated neutrophil recruitment, which can also help to alleviate inflammation [47,48].

The current scientific research has indicated that bromelain also exhibits notable mucolytic activity, making it a subject of interest in respiratory health. Its efficacy in reducing mucus production is linked to its ability to break down the glycoproteins present in mucus. By cleaving these proteins, bromelain effectively reduces the viscosity of mucus, facilitating more straightforward clearance from the respiratory tract. This process not only aids in alleviating the discomfort associated with excessive mucus production but also improves respiratory function. Moreover, the mucolytic action of bromelain has been studied in various clinical contexts, such as chronic bronchitis and chronic obstructive pulmonary disease, suggesting its potential as a therapeutic agent for conditions characterized by mucus hypersecretion [21,49,50]. The intricate interplay between bromelain’s enzymatic activity and the mucus composition underscores its role as a promising mucolytic agent, with implications for respiratory health and disease management. Overall, bromelain’s anti-inflammatory and immune system-modulating properties can help to reduce the respiratory tract’s swelling, inflammation, and mucus secretions, making breathing easier and more comfortable for those affected by upper respiratory tract infections, particularly in children. These observations represented the rationale for the use of bromelain to counteract the inflammatory processes related to upper respiratory tract infections in the present research. However, it is crucial to emphasize that extensive research and clinical trials are essential to validate the potential of this enzyme as a therapeutic agent in respiratory diseases, and it is worth further exploration.

## 5. Limitations of the Study

Although this study showed the effectiveness of bromelain supplementation in improving the symptoms associated with OME in children, it has some limitations. In particular, the retrospective design does not allow precise causality to be established between bromelain supplementation and the observed improvement in symptoms. The sample examined included only children in a limited age group (1–8 years), not allowing the results to be extended to other age groups or pediatric populations. Another limitation concerns the absence of long-term follow-up, which would have helped to assess the treatment’s persistence even after the end of the intervention. Finally, although the present study considered different variables, it would also have been appropriate to evaluate other factors, such as diet and environmental conditions, that might play a role in the mechanisms underlying drug absorption and recovery timing.

The results of this study need confirmation by future multicenter, prospective trials in order to establish the generalizability of our results. These trials would help to confirm whether bromelain supplementation could be generalized to a larger group of children and various clinical settings.

## 6. Conclusions

The present research sought to evaluate how supplementation with products of natural origin could be a valuable support for standard drug therapies. Specifically, this study demonstrated that adding bromelain to the standard drug therapy significantly improved the clinical outcomes of children with OME compared to those who received only fluticasone and saline. Bromelain’s potential as an effective adjunct to conventional treatments is highlighted by its ability to reduce middle ear mucus production and improve tympanic membrane mobility, demonstrated by the tympanometry results. Moreover, the considerable increase in otoacoustic emission and audiometric performance in the supplementation group compared to the control group highlights the role of bromelain in improving hearing function associated with OME. These results contribute to the knowledge of bromelain’s anti-inflammatory and mucolytic properties, highlighting its potential use as a valuable support for conventional drugs. Adding bromelain to standard therapy could significantly reduce the recovery time and exposure to synthetic drugs and could be an exciting starting point for further study.

In conclusion, our results have shown that integrating bromelain into a standard protocol could represent a significant breakthrough in audiology, offering a safer strategy that is effective and supplemental to conventional drug therapy for upper respiratory infections.

Understanding the actual clinical efficacy of natural products is of fundamental importance. It can encourage the creation of new therapeutic strategies to resolve different diseases by minimizing the healing times and possible collateral effects induced by administering the most common synthetic drugs. Natural products generally have a good safety profile, although they can cause adverse reactions or interactions with other medications. Therefore, it is always advisable to use them responsibly and under the supervision of a health professional. It is essential to point out that, although natural supplements can offer exciting opportunities for the curing of diseases, rigorous scientific research is needed to assess their effectiveness, safety, and clinical applications.

Bromelain supplementation may be particularly recommended in clinical situations where standard treatments for OME, including decongestants or antibiotics, are unsatisfactory or contraindicated—for example, in children with recurrent OME, those at risk of antibiotic resistance, or in cases where the risks of side effects from prolonged standard treatment make it undesirable. Well-designed clinical trials and regulatory approval are essential to ensure the effectiveness and quality of natural products used in medical practice.

## Figures and Tables

**Table 1:** Correlation between data obtained from otoscopic evaluation with the parameters obtained from other audiological assessments used in this study. The data refer to 224 patients divided into the control group (CTG) *n* = 112 and the natural supplement control group (NSG) *n* = 112. * *p* <0.05; ** *p* < 0.01.

	CTR			NSG		
	Pearson r	*p* Values	CI (95%)	Pearson r	*p* Values	CI (95%)
Otoscopy	1.000	0.000	1.000–1.000	1.000	0.000	1.000–1.000
Tympanometry RE	0.269	0.004 **	0.08761–0.4328	0.240	0.011 *	0.05662–0.4071
Tympanometry LE	0.187	0.048 *	0.001412–0.3600	0.016	0.867	0.1701–0.2010
Otoacoustic Emissions RE	0.310	0.01 **	0.1322–0.4687	0.205	0.030 *	0.02066–0.3766
Otoacoustic Emissions LE	0.217	0.022 *	0.03279–0.3870	0.238	0.011 *	0.05490–0.4057
Audiometry	0.193	0.042 *	0.007578–0.3653	0.221	0.019 *	0.03731–0.3908

**Table 2:** Differences in the tympanogram type among the natural supplement group (NSG) and control group (CTG) in the two follow-up times. RE, right ear; LE, left ear. * *p* < 0.05; *** *p* < 0.001.

Tympanometry	T1 vs. T0	T2 vs. T1
Type B		
RE	χ^2^ = 12.10, z = 3.478, *p* = 0.0005 ***	χ^2^ = 2.526, z = 1.589, *p* = 0.1120
LE	χ^2^ = 29.86, z = 5.465, *p* < 0.0001 ***	χ^2^ = 0.1623, z = 0.4029, *p* = 0.6870
Type C		
RE	χ^2^ = 2.233, z = 1.494, *p* = 0.1351	χ^2^ = 0.3958, z = 0.6292, *p* = 0.5292
LE	χ^2^ = 5.734, z = 2.395, *p* = 0.0166 *	χ^2^ = 0.5492, z = 0.7411, *p* = 0.4586

**Table 3:** Data show the differences between the supplement group (NSG) and the control group (CTG) in the number of patients with TEOAEs in each ear. All data refer to the two follow-up times. TEOAEs: transient-evoked otoacoustic emissions; RE, right ear; LE, left ear. ** *p* < 0.01; *** *p* < 0.001.

TEOAEs	T_1_ vs. T_0_	T_2_ vs. T_1_
Refer		
RE	χ^2^ = 17.78, z = 4.217, *p* < 0.0001 ***	χ^2^ = 0.1642, z = 0.4053, *p* = 0.6853
LE	χ^2^ = 7.238, z = 2.690, *p* = 0.0071 **	χ^2^ = 0.3368, z = 0.5804, *p* = 0.5617
Pass		
RE	χ^2^ = 9.061, z = 3.010, *p* = 0.0026 **	χ^2^ = 3.337, z = 1.827, *p* = 0.0677
LE	χ^2^ = 0.02165, z = 0.1471, *p* = 0.8830	χ^2^ = 8.180, z = 2.860, *p* = 0.0042 **

## Data Availability

The data presented in this study are available on request from the corresponding author due to legal or ethical reasons.
